# Functional Characterization of *ABCA4* Missense Variants Aids Variant Interpretation and Phenotype Prediction in Patients With ABCA4-Retinal Dystrophies

**DOI:** 10.1167/iovs.65.10.2

**Published:** 2024-08-01

**Authors:** Sigrid Aslaksen, Ingvild Aukrust, Laurie Molday, Josephine Prener Holtan, Ragnhild Wivestad Jansson, Siren Berland, Eyvind Rødahl, Cecilie Bredrup, Ragnheiður Bragadóttir, Eirik Bratland, Robert S. Molday, Per Morten Knappskog

**Affiliations:** 1 Department of Clinical Science, University of Bergen, Bergen, Norway; 2 Department of Medical Genetics, Haukeland University Hospital, Bergen, Norway; 3 Department of Biochemistry and Molecular Biology, University of British Columbia, Vancouver, BC, Canada; 4 Department of Ophthalmology, Oslo University Hospital, Oslo, Norway; 5 Department of Ophthalmology, Haukeland University Hospital, Bergen, Norway; 6 Department of Clinical Medicine, University of Bergen, Bergen, Norway; 7 Institute of Clinical Medicine, University of Oslo, Oslo, Norway; 8 Department of Ophthalmology and Visual Sciences, University of British Columbia, Vancouver, BC, Canada

**Keywords:** inherited retinal dystrophies (IRDs), ABCA4, phenotype-genotype, functional characterization, classification of ABCA4 variants

## Abstract

**Purpose:**

Biallelic pathogenic variants in the gene encoding the ATP-binding cassette transporter ABCA4 are the leading cause of irreversible vision loss in inherited retinal dystrophies (IRDs). Interpretation of *ABCA4* variants is challenging, due to *cis*-modifying and hypomorphic variants. We have previously detected 10 missense variants of unknown significance (VUS) in patients with suspected ABCA4-retinal dystrophies (ABCA4-RDs) in Norway. In this study, we functionally characterized the VUS to aid interpretation of the variants and to determine if they are associated with the disease.

**Methods:**

The *ABCA4* VUS were expressed in HEK293T cells and the ABCA4 expression level and ATPase activity were determined and correlated with the patients’ phenotype. The functional data further used for reclassification of the VUS following the American College of Medical Genetics and Genomics (ACMG) guidelines.

**Results:**

Of the 10 VUSs, 2 variants, Cys205Phe and Asn415Thr, were categorized as functionally severe. The age at presentation in the 2 patients carrying these variants was divergent and seemed to be driven by the patients’ second pathogenic variants Gly1961Glu and c.5461-10T>C, respectively. Three variants, Val643Gly, Pro799Leu, and Val1433Ile were categorized as functionally moderate, and were found in patients with intermediate/late age at presentation. The remaining five variants were categorized as functionally normal/mild. Based on our data, c.614G>T p.(Cys205Phe), c.1244A>C p.(Asn415Thr), and c.2396C>T p.(Pro799Leu) were reclassified to (likely) pathogenic, while 4 of the functionally normal/mild variants could be reclassified to likely benign.

**Conclusions:**

Functional analyses of *ABCA4* variants are a helpful tool in variant classification and enable us to better predict the disease severity in patients with ABCA4-RDs.

Inherited retinal dystrophies (IRDs) are a group of disorders that lead to severe and irreversible vision loss among children and young adults. Pathogenic variants in *ABCA4* have now been recognized as a major global cause of IRDs, accounting for 30% of all autosomal recessive IRDs^1^ and giving rise to a broad spectrum of phenotypes termed ABCA4-retinal dystrophies (ABCA4-RDs). Stargardt disease (STGD1:OMIM#248200) is the most common form of ABCA4-RD and is characterized by atrophy of both retinal pigment epithelium (RPE) cells and the overlying photoreceptors, with yellow-white flecks around the macula and mid-peripheral region. Patients with Stargardt disease develop central visual field loss, reduced visual acuity, impaired color vision, and delayed dark adaptation.^2^

*ABCA4* is a large gene comprising 50 exons and 2273 amino acids.^3^ The encoded protein belongs to the A-subfamily of ATP-binding cassette (ABC) transporters and is expressed in rod and cone photoreceptors. Here, it plays an important role in flipping *N*-retinylidene-phosphatidylethanolamine (*N-*Ret-PE), the Schiff base conjugate of retinal and the phospholipid phosphatidylethanolamine (PE), across disc membranes in the outer segments of photoreceptors. If *N*-Ret-PE is not efficiently transported out by ABCA4, it reacts with a second retinal molecule to form toxic bis-retinoids.^4^ Accumulation of these compounds over time will lead to RPE cell death followed by degeneration of photoreceptors and vision loss.^2^

More than 2200 *ABCA4* variants have been reported, where 60% are missense variants and the rest being nonsense variants, frameshifts, deletions, insertions, and (deep) intronic variants giving rise to splice defects.^2^ There is a genotype-phenotype correlation for ABCA4-RD, the genetic complexity makes it challenging to predict the clinical outcome of varying variant combinations.^5^ Nevertheless, a recently reported genotype-phenotype correlation matrix demonstrated a clear correlation when using a genotype classification system based on the presence of hypomorphic, moderate, severe or PVS1 (causing loss of function effect) variants.^6^ This genotype classification has further been validated and supported in several other recent publications focusing on genotype-phenotype correlations, highlighting that genotypic groups indeed influence the rate of disease progression.^7^^,^^8^ However, some heterogeneity in the phenotypic staging was found among patients carrying missense variants due to variability in residual protein function. Therefore, to better differentiate within the missense group, it is crucial to perform functional studies to determine whether a missense variant falls into the severe or moderate category. Such studies will enable a more precise genotype classification that better predict the clinical severity and disease progression in patients with ABCA4-RD.

Genetic screening of patients with suspected ABCA4-RDs often reveals *ABCA4* variants of unknown significance (VUS), some of which have not been previously reported. Interpreting the effect and contribution of each *ABCA4* VUS in disease development is often difficult, as patients frequently carry more than two variants, including *cis*-acting modifiers and hypomorphic variants.^5^^,^^6^^,^^9^ An important tool to aid variant interpretation is functional analyses, which are implemented as strong criteria either in pathogenic or benign direction in the original American College of Medical Genetics and Genomics and the Association for Molecular Pathology (ACMG-AMP) guidelines for variant interpretation.^10^ In our previous study on the genetics of patients with suspected ABCA4-RDs in Norway,^11^ approximately 32% (*n* = 27) of all detected *ABCA4* variants were classified as VUS. Following the exclusion of hot (closer to likely pathogenic), cool and cold (closer to likely benign) VUS,^12^ 10 missense variants found in 10 patients remained. The purpose of this study was to (i) analyze the biochemical and functional effects of these 10 missense VUS, (ii) correlate the functional data with the clinical phenotype of the patients, and then (iii) use the functional data to reclassify the VUS following the ACMG-AMP guidelines.

## Materials and Methods

### Clinical Assessment and Genotyping of Patients

The 10 missense VUS were found in 10 patients with an IRD consistent with an ABCA4-RD phenotype. Two patients were monozygotic twins and therefore only nine patients were genetically unique. Six patients were clinically assessed at the Department of Ophthalmology, Haukeland University Hospital, Bergen and four patients at the Department of Ophthalmology, Oslo University Hospital, Norway. Clinical data, obtained from the patients’ medical records, are listed in Table 1 and include clinical diagnosis, age at presentation, best corrected visual acuity (BCVA), full-field electroretinography (ffERG) recordings, optical coherence tomography (OCT) and ultra-widefield fundus autofluorescence (UWF-FAF) images. The patients’ genotype, phenotype stage, age at presentation, and disease duration are listed in Table 2. The staging was determined according to the staging system described by Klufas et al.^11^^,^^13^ Stages I–II are defined as central atrophy, only flecks (no atrophy) extending beyond the posterior pole (55° field view). Stage IIIA is defined as central atrophy and scattered atrophy extending the posterior pole. Stage IIIB-C is defined as central atrophy and severe atrophy extending from the macula to the equator.^11^ The 10 missense VUS listed in Table 3, were detected by either Sanger sequencing or next-generation sequencing (NGS) including a gene panel containing 268 genes involved in IRD, as previously described.^11^ Patients 9 and 10 have also been re-analyzed with an updated NGS-retina panel including 334 genes involved in IRD (a list of genes is available at www.genetikkportalen.no) and both these patients have also been analyzed by whole-genome sequencing (WGS; with special focus on *ABCA4*).

**Table 1.** Clinical Characteristics of the 10 Patients Investigated in this Study

				BCVA			
Study No (Family ID)	Age at Presentation	Disease Duration	Stage^†^	Right	Left	Electroretinography	Phenotype
P1 (1)	16	14	I	20/125	20/125	Normal ffERG. Central macular dysfunction on mfERG	Visual loss, photophobia and a small central scotoma. Central macular atrophy without
P2 (1)	16	14	I	20/200	20/125		foveal sparing
P3^*^	31	NA	NA	NA		NK	Hyperfluorescent ring in the central and midperipheral area. Loss of color vision. Genetic screening revealed *EYS-*mediated IRD
P4	36	25	I	20/100	20/100		Non-progressing bull's eye maculopathy
P5	6	43	IIIC	CF 30 cm	CF 30 cm	Rod-cone dysfunction	Rapid visual loss and nyctalopia. Confluent atrophy of the posterior pole and decreasing atrophic changes towards the far retinal periphery
P6	60	2	II	20/100	20/40		Developed cataracts (not operated) at age 73 y. Retina within the posterior pole was affected. Not possible to obtain new images due to unclear lens
P7	46	5	IIIA	20/20	20/25		Foveal sparing with parafoveal atrophy
P8	10	38	IIIB	20/400	CF 40 cm	Rod-cone dysfunction	Rapid visual loss and gradual nyctalopia. Patchy macular atrophy without foveal sparing. Reticular atrophy extending beyond the arcades with retinal vessel attenuation, but better-preserved peripheral retina
P9	19	38	IIIB	20/400	CF 100 cm	Rod-cone dysfunction	Gradual vision loss and increasing nyctalopia. Extensive chorioretinal atrophy of the posterior pole without foveal sparing and reticular atrophy extending toward the periphery
P10	60	NA	NA	20/40	20/63	Rod-cone dysfunction	Nyctalopia and paracentral visual field loss. Midperipheral yellow flecks and confluent posterior pole atrophy extending peripherally, but with foveal sparing (not typical for ABCA4-RD). Progressive neurological hearing loss

ABCA4-RD = ABCA4-retinal dystrophies; BCVA = best corrected visual acuity; CF = counting fingers; ffERG = full*-*field electroretinogram; IRD = inherited retinal dystrophies; mfERG = multifocal electroretinogram; NA = not applicable; NK = not known.

* Because P3 has EYS-mediated cone dystrophy we did not include disease duration, staging, and BCVA for this patient.

† Stages I and II are defined as central atrophy, only flecks (no atrophy) extending beyond the posterior pole (55 degrees field view). Stage IIIA is defined as central atrophy and scattered atrophy extending the posterior pole. Stage IIIB-C is defined as central atrophy and severe atrophy extending from the macula to the equator.

**Table 2.** Genotype and Phenotype Characteristics of the 10 Patients Carrying the 10 ABCA4 VUSs (Marked in Bold) Investigated in this Study

Study No (Family ID)	Variant 1	Variant 2	Variant 3	Parental/Maternal Origin	Stage/Age at Presentation/Disease Duration
P1 (1)	**c.317A>T p.(Tyr106Phe)**	—	—	NA	I/16/14
	**—**	c.868C>T p.(Arg290Trp)	—	NA	
	**—**	—	c.2875A>G p.(Thr959Ala)	NA	
P2 (1)	**c.317A>T p.(Tyr106Phe)**	—	—	NA	I/16/14
	**—**	c.868C>T p.(Arg290Trp)	—	NA	
	**—**	—	c.2875A>G p.(Thr959Ala)	NA	
P3^*^	**c.514G>A p.(Gly172Ser)**	—	**—**	Maternal	/31/
	**—**	**c.6148G>C p.(Val2050Leu)**	**—**	Paternal	
P4	**c.614G>T p.(Cys205Phe)**	—	—	NA	I/36/25
	**—**	c.5882G>A p.(Gly1961Glu)	—	NA	
	**—**	—	c.3523-9C>G	NA	
P5	**c.1244A>C p.(Asn415Thr)**	—	—	NA	IIIC/6/43
		c.5461-10T>C p.Thr1821Aspfs^*^6	—	NA	
	**—**	—	c.5603A>T p.(Asn1868Ile)	NA	
P6	**c.1928T>G p.(Val643Gly)**	—	—	NA	II/60/2
	**—**	c.6449G>A p.(Cys2150Tyr)	—	NA	
P7	**c.2396C>T p.(Pro799Leu)**	—	—	NA	IIIA/46/5
	**—**	c.4734del p.(Leu1580^*^)	—	NA	
P8	**c.2819C>G p.(Pro940Arg)**	—	—	Maternal	IIIB/10/38
	**—**	c.3364G>A p.(Glu1122Lys)	—	Maternal	
	**—**	—	c.161-23T>G p.(Cys54=, Cys54Serfs^*^14)	Paternal	
P9	**c.3491A>G p.(Lys1164Arg)**	—	—	Maternal	IIIB/19/38
	**—**	c.769-784C>T p.(Leu257=, Leu257Aspfs^*^3)	—	Maternal	
	**—**	—	c.4540-2077C>T	Paternal	
P10	**c.4297G>A p.(Val1433Ile)**	—	—	NA	/60/
		c.3607+771G>A	—	NA	

NA = not available.

* For P3, only maternal samples were available, and, for P8 and P9, only paternal samples were available. We can assume that the variants not present in the parents tested are found on the opposite allele (de novo variants in *ABCA4* are less likely). For the rest of the patients, parental samples were not available. Definitions of stages I–III can be found in the “Materials and Methods” section. The ACMG-AMP classifications of all the variants are given in Table 4. Reference transcript: NM_000350.3 (*ABCA4*).

**Table 3.** Overview of Functional Data and Predicted Severity of *ABCA4* Missense VUS Detected in the 10 Patients Investigated in this Study

Variant	Relative Expression Levels ± SD	Intracellular Localization	Relative Basal ATPase Activity ± SD	Relative ATR Induced ATPase Activity ± SD	F-Index	Predicted Severity
WT	100	Vesicles	100	171 ± 14	1.00	Normal
c.317A>T p.(Tyr106Phe)	111 ± 10	Vesicles	95 ± 10	153 ± 14	0.94	Normal/mild
c.514G>A p.(Gly172Ser)	113 ± 6	Vesicles	100 ± 4	183 ± 13	1.35	Normal/mild
c.614G>T p.(Cys205Phe)	50 ± 11	ER/Vesicles	41 ± 10	42 ± 1	0.00	Severe
c.1244A>C p.(Asn415Thr)	72 ± 11	Vesicles	58 ± 9	56 ± 10	−0.02	Severe
c.1928T>G p.(Val643Gly)	121 ± 12	Vesicles	83 ± 6	115 ± 15	0.48	Moderate
c.2396C>T p.(Pro799Leu)	43 ± 5	ER/small vesicles	70 ± 13	99 ± 16	0.17	Moderate
c.2819C>G p.(Pro940Arg)	100 ± 18	Vesicles	100 ± 7	170 ± 20	1.01	Normal/mild
c.3491A>G p.(Lys1164Arg)	115 ± 23	Vesicles	93 ± 4	155 ± 30	0.90	Normal/mild
c.4297G>A p.(Val1433Ile)	86 ± 12	Vesicles	76 ± 17	112 ± 28	0.41	Moderate
c.6148G>C p.(Val2050Leu)	107 ± 13	Vesicles	98 ± 10	157 ± 3	0.92	Normal/mild

All patients provided signed informed consent to participate in the study. The study was approved by the Regional Ethics Committee (case no. 2018/507/REC South-East). Patients who were examined with WGS signed an additional informed consent following approval from the Regional Ethics Committee (case no. 2017/2487/REC West). The study followed the tenets of the Declaration of Helsinki.

### Creating a Model Based on Cryo-EM and AlphaFold

**Figure 1.**
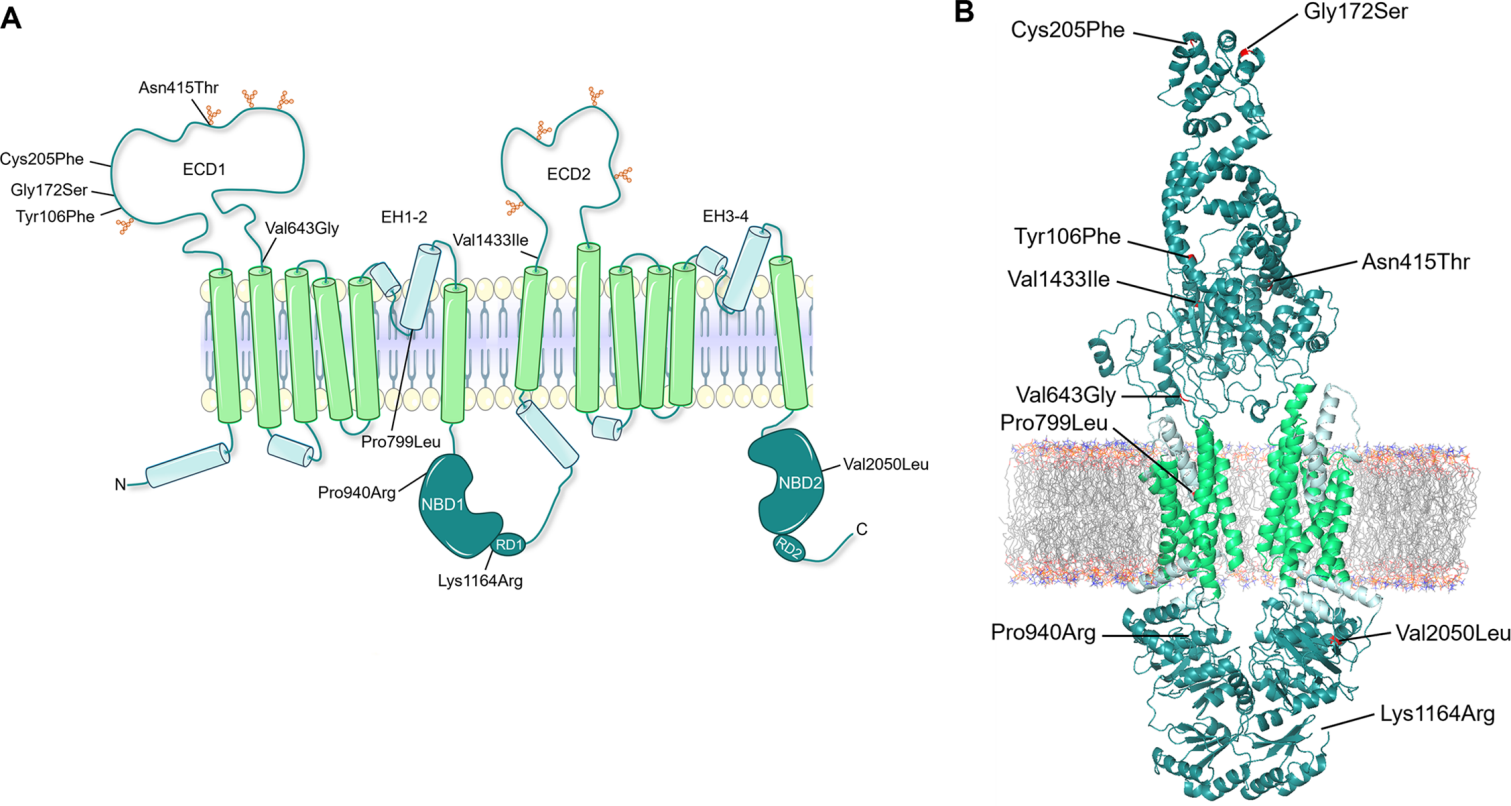
(**A**) Topological models of ABCA4 showing the locations of the 10 missense variants examined in this study. There are two nonidentical tandem halves of ABCA4, each containing a N-glycosylated exocytoplasmic domain (ECD), a transmembrane domain (TMD) consisting of 6 transmembrane segments, a nucleotide-binding domain (NBD), and a regulatory domain (RD). (**B**) Structure of ABCA4 based on the sequence of ABCA4, the cryoEM structure of ABCA4 in its unbound state, and the AlphaFold model.

The ABCA4 structure model (Fig. 1B) was created by making a homology model based on the sequence of ABCA4 and the cryo-EM structure of ABCA4 in its unbound state (PDB: 7M1P, https://www.rcsb.org/structure/7M1P). The amino acids 111-285 from the exocytoplasmic domain 1 (ECD1) of the AlphaFold model (PDB: P78363, https://alphafold.ebi.ac.uk/entry/P78363) were then transferred to the homology model to create a “hybrid” homology model.

### Generation of DNA Constructs

The 10 missense VUS were generated by site-directed mutagenesis (Agilent, Santa Clara, CA, USA) in pCEP4 vector encoding the human *ABCA4* (NM_000350) containing a C-terminal 1D4 tag (TETSQVAPA), as previously described.^14^ This tag has proven to be very effective for immunoaffinity purification of membrane proteins and does not disturb the biochemical properties of ABCA4.^14^ The *ABCA4* sequence of the 10 different plasmids was fully sequenced to verify the mutagenesis.

### Expression of ABCA4 Variants in HEK293T Cells

HEK293T cells were transfected when 80% confluent in 6-well plates with 1 µg of pCEP4-ABCA4-1D4 using 1 mg/mL PEI MAX (Polysciences, Warrington, PA, USA) at a ratio of 3:1 of PEI to DNA. At 24 hours post-transfection, the cells were harvested and centrifuged at 1500 rpm for 4 minutes. The cell pellet was resuspended in 20 µL resuspension buffer (50 mM HEPES, 150 mM NaCl, 5 mM MgCl_2_, 10% glycerol, and pH 7.4), solubilized for 30 minutes at 4°C in 125 µL solubilization buffer (20 mM CHAPS, 50 mM HEPES, 150 mM NaCl, 5 mM MgCl_2_, 10% glycerol, 1 mM DTT, 1x Protease Inhibitor Cocktail [PIC] [Calbiochem, Merck KGaA, Darmstadt, Germany], and pH 7.4). To keep the mutants in a native-like state, we used the mild detergent CHAPS when solubilizing the cell pellets. The samples were then centrifuged at 40,000 rpm for 12 minutes in a TLA55 rotor using a Beckman Optima TL centrifuge. Supernatants were then run on an 8.5% SDS-PAGE gel followed by Western blotting to detect ABCA4-1D4 using Rho1D4 mouse monoclonal antibody (diluted 1:500)^14^ from the University of British Columbia (UBC) (https://ubc.flintbox.com/technologies/0f1ef64b-fa5d-4a58-9003-3e01f6f672a6). Anti-Ezrin antibody (rabbit polyclonal; catalog no. ab235927; Abcam, Boston, MA, USA) was used as a loading control. All data presented are expressed as means ± SD for at least three independent experiments.

### Intracellular Localization of ABCA4 Variants by Immunofluorescence Microscopy

Hela cells were seeded in a 6-well plate containing coverslips coated with poly-L-lysine and transfected for 48 hours, as described above. Next, the cells were fixed with 4% paraformaldehyde in 0.1 M phosphate buffer (PB) for 15 minutes, washed 3 times with PB, and blocked and permeabilized with 10% goat serum and 1% Triton X-100 in PB for 15–30 minutes. The coverslips were incubated with the Rho1D4 antibody (1:50) and rabbit polyclonal antibodies against calnexin (1:1000, catalog no. ab12504; Abcam, Boston, MA, USA) as an endoplasmic reticulum (ER) marker in labeling buffer consisting of 3% goat serum and 0.05% Triton X-100, for 1–2 hours at room temperature (RT). The coverslips were then washed 3 times with PB followed by labeling with secondary antibody using Alexa-488 goat-anti-mouse Ig (for ABCA4-1D4), Alexa594 goat-anti-rabbit Ig (for calnexin), and DAPI (for nuclei) in labeling buffer for 1 hour in the dark. The coverslips were washed 3 times with PB, mounted with Mowiol and visualized under a Zeiss LSM700 confocal microscope (Oberkochen, Germany) using a 40 × oil immersion objective (aperture of 1.3). Images were analyzed using Zeiss Zen software.

### ATPase Activity Assay

The ATPase activity assay was done as previously described.^15^^,^^16^ HEK293T cells were transfected in 10 cm plates at 80% confluency, as described above. The number of 10 cm plates needed for each variant was determined according to their expression levels. At 48 hours post-transfection, the cells were harvested and resuspended in 100 µL resuspension buffer (described above) per 10 cm plate. Samples were solubilized for 30 minutes at 4°C in 400 µL of column buffer (CB; 20 mM CHAPS, 50 mM HEPES, 150 mM NaCl, 5 mM MgCl_2_, 10% glycerol, 0.15 mg/mL brain polar lipid [BPL], and 0.05 mg/mL 1,2-dioleoyl-sn-glycero-3-phosphoethanolamine [DOPE]; Avanti Polar Lipids, Alabaster, AL, USA; 1 mM DTT, 1x PIC, and pH 7.4), and centrifuged at 40,000 rpm (corresponding to 100,000 × g) for 12 minutes in a TLA55 rotor or a TLA110.4, depending on the sample volumes, as described above. The supernatant was collected and incubated with approximately 80 µL Rho1D4-Sepharose affinity matrix per variant for 1 hour at 4°C. The beads were then washed three times in CB, transferred to a Millipore Ultrafree centrifugal filter unit, and washed three additional times. ABCA4-1D4 was eluted twice using 65–70 µL of CB containing 0.3 mg/mL of 1D4 peptide for 30 minutes at 18°C with shaking. To estimate the protein concentration in each sample, we ran an aliquot of each sample along with BSA standards on a gel and stained the protein bands using Coomassie blue.

The ATPase activity of each sample was measured using the ADP-Glo Kinase Assay (Promega, Madison, WI, USA), as previously described.^15^ For each variant, 15 µL (approximately 100–300 ng) of purified ABCA4 was incubated with either 1 µL of 0.8 mM all-trans retinal (ATR; Sigma-Aldrich, Oakville, ON, Canada) diluted in CB or 1 µL of CB for 20 minutes. By diluting ATR in CB containing PE, *N*-ret-PE could be formed. Four µL of 2.5 mM ultrapure ATP was then added for 35 minutes at 37°C. Six µL of each sample was then mixed with 6 µL ADP-Glo and incubated in the dark for 1 hour to terminate the reaction and deplete any remaining ATP. The ATPase activity of ABCA4 was then measured by adding 12 µL of detection reagent for 35 minutes and recording the luminescence signal using SoftMax Pro 5.4 on a Molecular Device SpectraMax M3 Spectrometer. The ATP background signals for each variant were determined by having a separate 0 hour ATP reaction, where ATP was immediately mixed with 6 µL ADP-Glo. All data presented are expressed as means ± SD for at least three independent experiments.

### Functional Index 

The functional index (F-index) is a well-established parameter used to estimate the functional consequence of more than 70 *ABCA4* missense variants.^2^ To obtain an F-index of each variant, we multiplied the variants’ relative expression level (E) by their relative ATR-stimulated ATPase activity (S) (F = E x S).^2^ E is calculated by dividing the variant's expression level by the wild type’s (WT's) expression level. S is determined by dividing the ATR-stimulated activity minus the basal activity of the variant by the WT's ATR-stimulated activity minus the WT's basal activity. Variants with F-indexes close to 0 lack expression and/or ATPase activity and can be categorized as functionally severe. Variants with F-indexes above 0.7 and close to 1, where 1 represents WT ABCA4, can be categorized as functionally normal/mild. In between are the F-indexes ranging from 0.15 to 0.50, representing functionally moderate variants.

### Reclassification of *ABCA4* Variants

The *ABCA4* VUS detected in the 10 patients were reclassified after the functional analyses using the ACMG-AMP guidelines for variant interpretation.^10^ PM2 was used at the supporting level, as suggested by ClinGen Sequence Variant Interpretation general recommendations for using the ACMG-AMP criteria, a maximum of 3 homozygous individuals were accepted in GnomAD version 4.0.0 for the use of PM2. In patients with conflicting evidence in both the pathogenic and benign directions, we combined the evidence as suggested by Garrett et al. (2020) and ignored PM2_sup when this was the only evidence in the pathogenic direction.^17^ PM3 was used as suggested by ClinGen. For PVS1, we used the decision tree, as previously described.^18^ For the use of PP3/BP4 for missense variants, we used a REVEL cutoff of 0.7 and 0.4, respectively, and for intronic variants we used a SpliceAI cutoff of <0.2 and no evidence of prediction of novel exonic/deep intronic splice site of any strength (by SpliceSiteFinder and MaxEntScan) for the use of BP4. For the use of PM5, the variant under examination must have been reported previously in a patient with ABCA4-RDs, had been classified as P/LP in ClinVar, and have an equivalent or higher REVEL score than the reference variant OR both the reference variant and the variant under examination must have REVEL scores of ≥0.7. PS3 was used for variants with F-index <0.5, and BS3_sup was used for variants with F-index >0.9.

## Results

### Clinical Assessment of the 10 Patients Carrying the *ABCA4* VUS

All clinical data of the 10 patients are listed in Table 1.

### Molecular Modeling of the ABCA4 Variants

As shown in Figure 1, five of the variants are located in ECD1 (Tyr106Phe, Gly172Ser, Cys205Phe, Asn415Thr, and Val643Gly), one in transmembrane domain 1 (TMD1) (Pro799Leu), one in nucleotide binding domain 1 (NBD1) (Pro940Arg), one in regulatory domain 1 (RD1) (Lys1164Arg), one in ECD2 (Val1433Ile), and one in NBD2 (Val2050Leu). Since the residue Cys205 is part of a flexible region and not part of the cryo-EM structure, we transferred the amino acid residues 111-285 from ECD1 of the AlphaFold model to create a “hybrid” ABCA4 model.

### Expression of ABCA4 Variants

**Figure 2.**
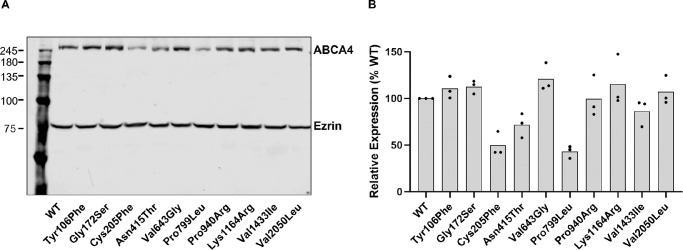
Protein expression of *ABCA4* missense variants relative to WT in transiently transfected HEK293 cells. (**A**) A representative Western blot labeled with Rho 1D4 antibody targeting WT ABCA4 and variants containing a 1D4 tag. Ezrin was used as a loading control for normalization. (**B**) Quantification of variants relative to WT (set to 100%), shown as mean of relative expression (*n* = 3). Each *dot* represents independent experiments.

Expression levels of the 10 ABCA4 variants in transfected HEK293T cells were evaluated by Western blotting (Fig. 2). Of the 10 variants, Pro799Leu and Cys205Phe showed reduced expression levels at approximately 40% to 50% of the WT, respectively. The Asn415Thr variant was expressed at approximately 70% compared to WT. The remaining seven variants (Tyr106Phe, Gly172Ser, Val643Gly, Pro940Arg, Lys1164Arg, Val1433Ile, and Val2050Leu) displayed expression levels comparable to the WT.

### Intracellular Localization of the ABCA4 Variants in Hela Cells

**Figure 3.**
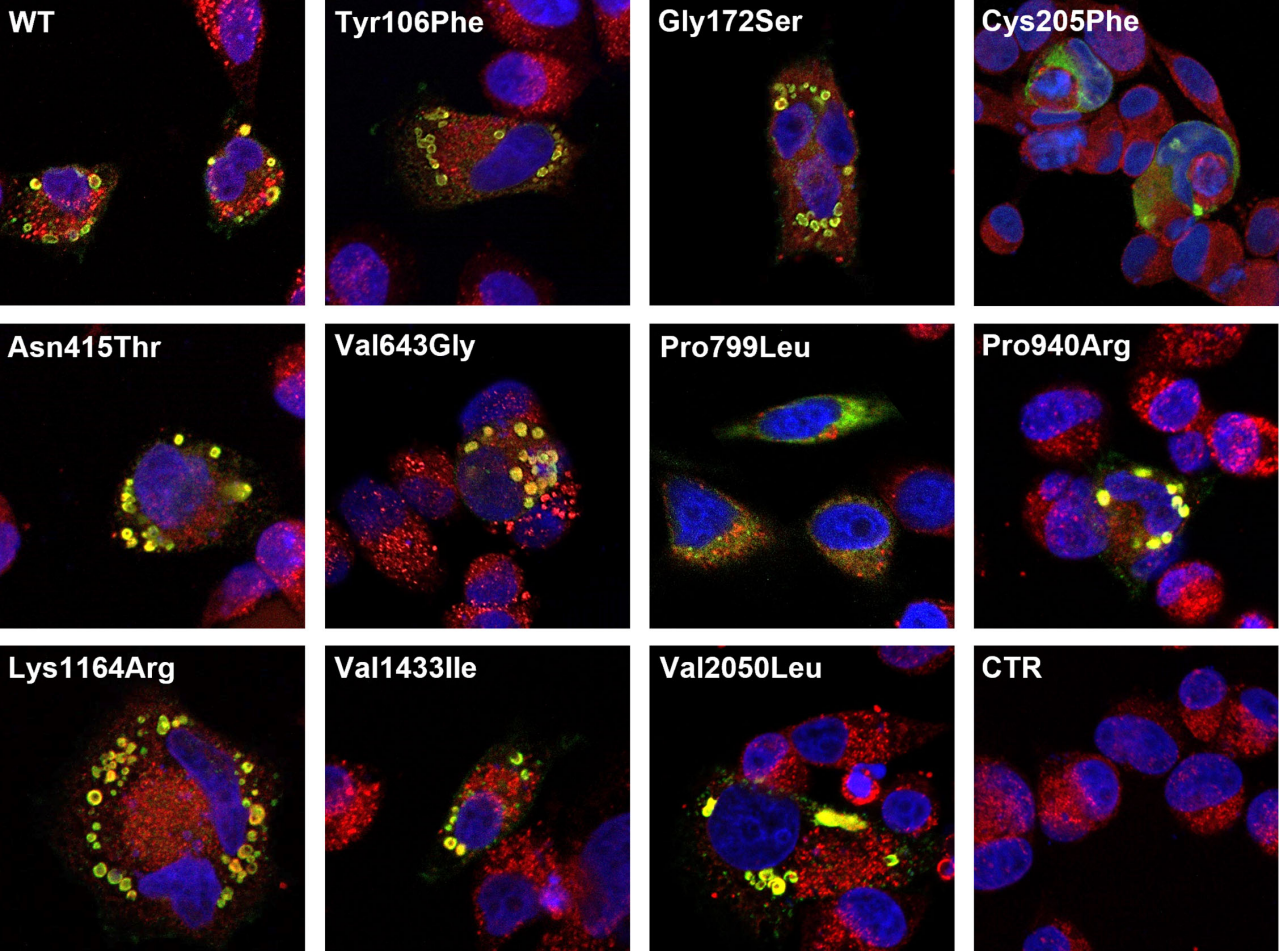
Intracellular localization of the ABCA4 variants in HeLa cells. Representative immunofluorescence images of transfected HeLa cells with plasmids encoding ABCA4 WT and variants or empty pCEP4 vector control (CTR). Rho1D4 antibody (*green*) was used to label ABCA4 containing the 1D4 tag, calnexin antibody (*red*) was used as an ER marker, and DAPI (*blue*) was used for nuclear counterstaining. The WT protein and variants expressed at WT levels localized to vesicles, whereas low-expressing variants (Cys205Phe and Pro799Leu) displayed reticular localization.

To determine the intracellular localization of the ABCA4 variants, we examined transiently transfected Hela cells by immunofluorescence analysis. According to previous studies, ABCA4 variants with solubilization and expression levels below WT ABCA4 tend to be retained in the ER, whereas ABCA4 variants that were expressed near WT levels localize more to intracellular vesicles.^15^^,^^16^ Of the 10 variants studied, 8 clearly localized to vesicles within or close to the ER (Fig. 3). This was expected as they were solubilized and expressed similarly to WT. The distribution of Cys205Phe, which was expressed at levels around 50% compared to WT, showed a 50/50 distribution in ER and vesicles. Pro799Leu, which displayed the lowest solubilization and expression level (approximately 40%) of all variants compared to WT, was the only variant with a clearly defined ER distribution and was only found in a few very small vesicles.

### ATPase Activity of the ABCA4 Variants

**Figure 4.**
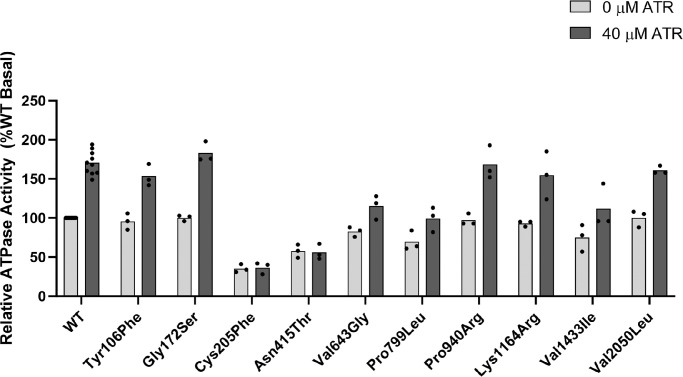
ATPase activity of purified ABCA4 WT and missense variants in the presence and absence of *N-*ret-PE. By diluting ATR in an assay buffer containing PE, *N*-ret-PE is formed. The basal ATPase activity of the variants is normalized to WT basal ATPase activity (set to 100%), and the retinal-stimulated activity of each variant is relative to their basal activity. *Bars* indicate the mean of three independent experiments, each represented by a *dot*.

To determine the *ABCA4* variants’ effect on the functional properties of ABCA4, we measured the ATPase activity for each variant in the presence and absence (basal activity) of *N*-ret-PE substrate. *N*-ret-PE forms when the aldehyde group of ATR reacts with the primary amine group of PEs in the buffer used in the ADP-Glo kinase assay. As shown in Figure 4, presence of *N*-ret-PE resulted in an average 1.7-fold increase in the WT activity, which is within the range of previous findings.^15^^,^^16^^,^^19^ Two of the variants, Cys205Phe and Asn415Thr, showed low basal activities compared to WT (approximately 40%-60%) with no increase in substrate-stimulated activity. The variants Val643Gly, Pro799Leu, and Val1433Ile exhibited close to WT basal activity, but reduced substrate-stimulated activity, indicating that these amino acid changes affect the ability of ABCA4 to bind to its substrate *N*-ret-PE. The remaining five variants (Tyr106Phe, Gly172Ser, Pro940Arg, Lys1164Arg, and Val2050Leu) showed no effect on either the basal- or substrate-induced activity.

### Categorizing the *ABCA4* Variants Using the F-Index

**Figure 5.**
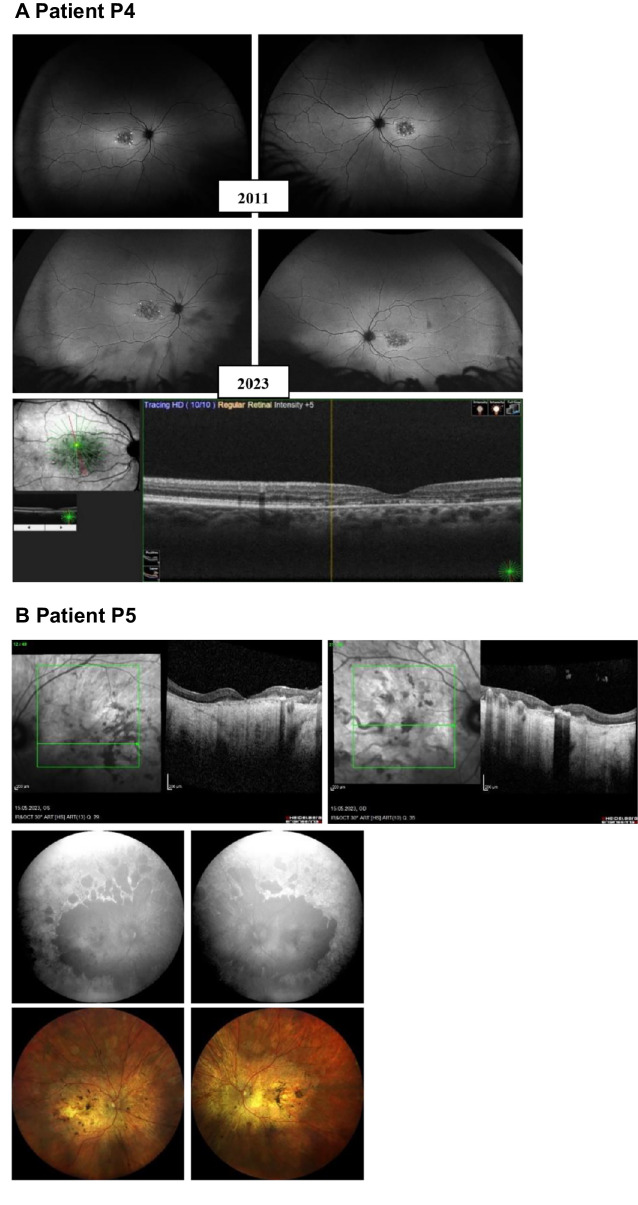
OCT and UWF-FAF images of P4 and P5 with functionally severe variants characterized in this study. (**A**) P4: UWF-FAF image and OCT showing degeneration in the macula with limited change over a 12-year period. No peripheral degeneration is present. Para-macular retinal function was present 25 years after the disease onset of symptoms. (**B**) P5: OCT image of the macula demonstrates the extensive chorioretinal atrophy with pigment deposition and no foveal sparing. UWF-FAF and color fundus images of the right and left eye highlight the confluent atrophy of the posterior pole and the peripheral degeneration.

**Figure 6.**
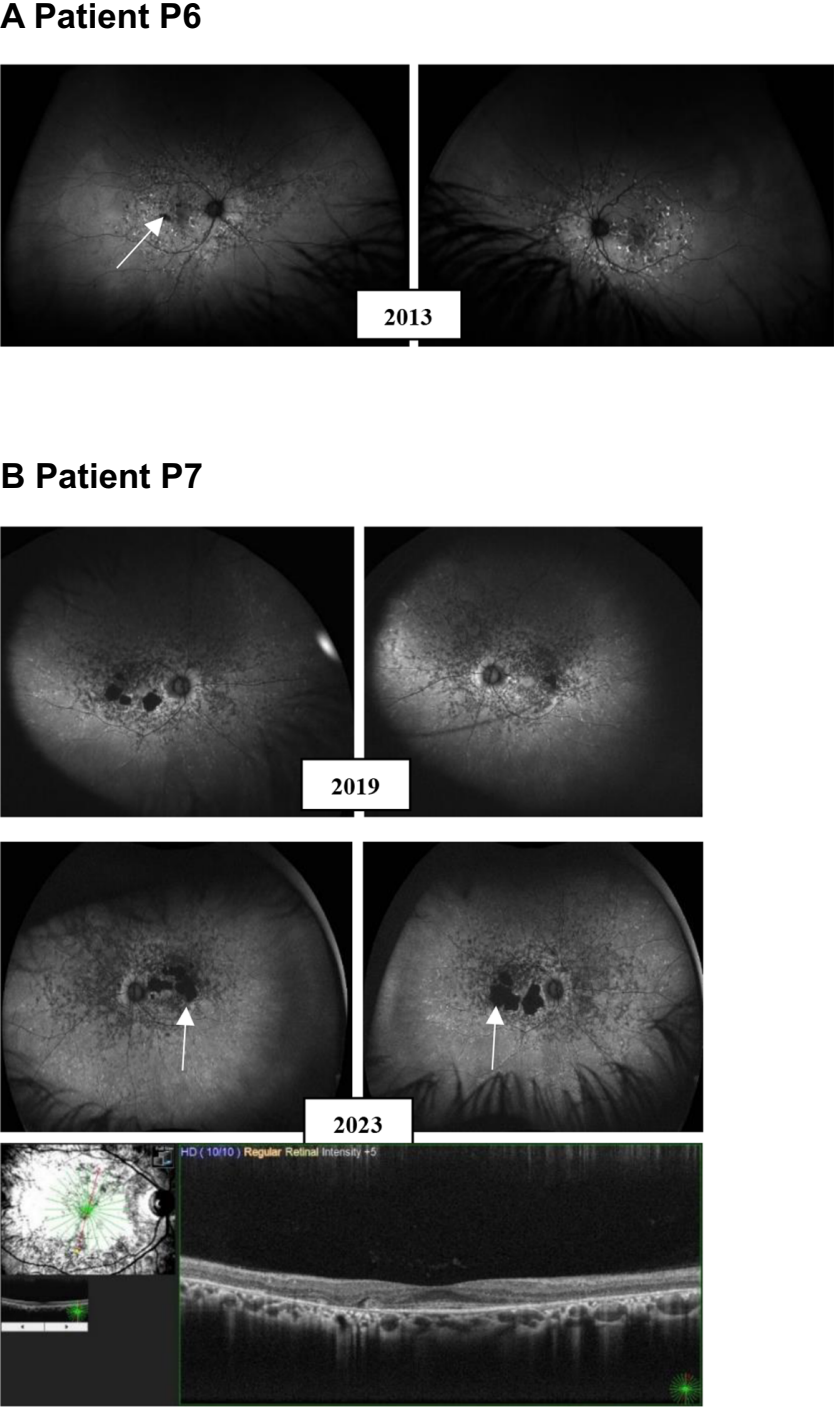
OCT and UWF-FAF images of P6 and P7 with functionally moderate variants characterized in this study. (**A**) P6: UWF-FAF image shows mid-peripheral changes, foveal sparing, and some areas with complete atrophy (*white arrow*), 2 years after onset of symptom. New images could not be obtained due to cataract. (**B**) P7: UWF-FAF and OCT images showing mid-peripheral and peripheral changes of the retina at the time of diagnosis (2019). Foveal sparing was preserved four years after diagnosis, however, increasing atrophy para-macular (*white arrow*).

To categorize the variants, we combined protein expression levels and ATPase activities to generate an F-index for each variant.^2^ As shown in Table 3, two variants, Cys205Phe and Asn415Thr, have F-indexes close to 0 and can be categorized as functionally severe. Three variants, Val643Gly, Pro799Leu, and Val1433Ile, have F-indexes of 0.48, 0.17, and 0.41, respectively, indicating functionally moderate variants. The last five variants, Tyr106Phe, Gly172Ser, Pro940Arg, Lys1164Arg, and Val2050Leu have F-indexes ranging from 0.90–1.35 and are considered normal/mild variants that are probably not associated with the patients’ phenotypes. Based on the functional data, we decided to present OCT and UWF-FAF images of the patients carrying the functionally severe (Fig. 5) and moderate variants (Fig. 6, Supplementary Fig. S1B).

### Reclassification of *ABCA4* Variants Using ACMG-AMP Classification System

The *ABCA4* VUS included in this study were reclassified after the functional analyses and the results are shown in Table 4. Variant c.614G>T p.(Cys205Phe) was reclassified as pathogenic, c.1244A>C p.(Asn415Thr) and c.2396C>T p.(Pro799Leu) were reclassified as likely pathogenic, while c.317A>T p.(Tyr106Phe), c.2819C>G p.(Pro940Arg), c.3491A>G p.(Lys1164Arg), and c.6148G>C p.(Val2050Leu) were reclassified as likely benign. The variants c.514G>A p.(Gly172Ser), c.1928T>G p.(Val643Gly), and c.4297G>A p.(Val1433Ile), however, could not be reclassified and remained as VUS.

**Table 4.** ACMG-AMP Classification of *ABCA4* Variants. The Variants Characterized in This Study Are Marked in Bold.

Patient ID	Nucleotide/Protein Variant	REVEL	SpliceAI	ACMG Subscores (After Functional Testing)	ACMG Classification (After Functional Testing)
P1, P2	**c.317A>T p.(Tyr106Phe)**	0.261	0.01	BS3_sup, BP4, PM2_sup	*Likely benign*
	c.868C>T p.(Arg290Trp)	0.525	0.06	PM3_very strong, PM5, PM2_sup	*Pathogenic*
	c.2875A>G p.(Thr959Ala)	0.962	0.00	PM3, PM5, PM2_sup, PP3	*Likely pathogenic*
P3	**c.514G>A p.(Gly172Ser)**	0.496	0.03	PM3_strong, PM2_sup, BS3_sup, BP2, BP5	*VUS*
	**c.6148G>C p.(Val2050Leu)**	0.795	0.01	BS3_sup, BP2, BP5, PP3	*Likely benign*
P4	**c.614G>T p.(Cys205Phe)**	0.967	0.00	PS3, PM2_sup, PM3, PM5, PP3, PP5	*Pathogenic*
	c.5882G>A p.(Gly1961Glu)	0.76	0.01	PM3_very strong^‡^, PM5, PP3, PP5	*Pathogenic*
	c.3523-9C>G	NA	0.02	PM2_sup, BP2, BP4	*Likely benign*
P5	**c.1244A>C p.(Asn415Thr)**	0.817	0.00	PS3, PM2_sup, PM3_sup, PP3	*Likely pathogenic*
	c.5461-10T>C p.Thr1821Aspfs^*^6	NA	0.07	PM3_very strong, PS3, PM2_sup, PP5	*Pathogenic*
	c.5603A>T p.(Asn1868Ile)	0.402	0.00	Hypomorphic	*NA*
P6	**c.1928T>G p.(Val643Gly)**	0.944	0.01	PS3, PP3	*VUS*
	c.6449G>A p.(Cys2150Tyr)	0.923	0.02	PM3_very strong, PM2_sup, PP3, PP5	*Pathogenic*
P7	**c.2396C>T p.(Pro799Leu)**	0.542	0.01	PS3, PM2_sup, PM3_sup	*Likely pathogenic*
	c.4734del p.(Leu1580^*^)	NA	0.01	PVS1, PM3, PM2_sup	*Pathogenic*
P8	**c.2819C>G p.(Pro940Arg)**	0.273	0.00	PM2_sup, BS3_sup, BP4, BP2	*Likely benign*
	c.3364G>A p.(Glu1122Lys)	0.939	0.02	PM3_very strong, PM2_sup, PP3, PP5	*Pathogenic*
	c.161-23T>G p.(Cys54=, Cys54Serfs^*^14)	NA	0.02	PM3_strong, PS3_mod, PM2_sup	*Likely pathogenic*
P9	**c.3491A>G p.(Lys1164Arg)**	0.199	0.01	PM2_sup, BS3_sup, BP4	*Likely benign*
	c.769-784C>T p.(Leu257=, Leu257Aspfs^*^3)	NA	0.08	PM2_sup, PS3_sup, PM3_strong, BP2	*VUS*
	c.4540-2077C>T	NA	0.00^†^	PM2_sup	*VUS*
P10	**c.4297G>A p.(Val1433Ile)**	0.605	0.10	PS3	*VUS*
	c.3607+771G>A	NA	0.00	PM2_sup, BP4	*VUS*

Reference transcript: NM_000350.3 (*ABCA4*).

† c.4540-2077C>T did not fulfill BP4 both SpliceSiteFinder and MaxEntScan indicate the activation of a cryptic acceptor site.

‡ For c.5882G>A p.(Gly1961Glu), we used PM3, even though PM2_sup is not met, this variant is known to give a mild late-onset phenotype. For intronic variants, we did not combine PS3 with PP3/BP4.

## Discussion

By combining functional and biochemical characterizations of *ABCA4* variants with in-depth clinical investigations of patients with IRDs, we here demonstrate the added value of functional data in genetic variant interpretation and classification of genotypes comprising missense variants. Using the approach outlined in the current article, we have successfully resolved the clinical significance of 7 of the 10 variants previously characterized as VUS.

Many variants have been reported in the *ABCA4* gene, and it is often challenging to determine if they are pathogenic (severe or moderate) or benign. To classify *ABCA4* variants, understand which variant is disease-causing, and predict the severity and progression of disease, it is crucial to understand the molecular effect of variants on the protein level. Our previous study detected 10 missense *ABCA4* variants that have never been functionally characterized before in 10 patients with suspected ABCA4-RDs. To determine if these VUS could be responsible for the patients’ IRD, we analyzed their effect on the protein expression level, the functional activity of ABCA4, and the intracellular localization. In accordance with previous studies, we used the variants’ solubilization levels in the mild detergent CHAPS as a measure of their expression levels.^2^^,^^16^ To study their functional activities, we measured how the basal ATPase activity was stimulated in the presence of *N*-Ret-PE, as this method has proven to be the most efficient way to determine the functional activity of ABCA4.^2^^,^^15^^,^^20^^,^^21^ Based on these data, we further determined the F-index of each of these variants to gain a better understanding of their effect on the patients’ phenotype. According to previous studies, there is a good correlation between the F-index and age at presentation, which generally correlates well with disease severity, at least when differentiating between severe and milder prognoses.^2^ However, the effect of a variant will always depend on the variant in *trans*, as the variant combination is what determines the phenotype of a patient. Therefore, the F-index of a variant cannot alone predict the phenotype.

**Figure 7.**
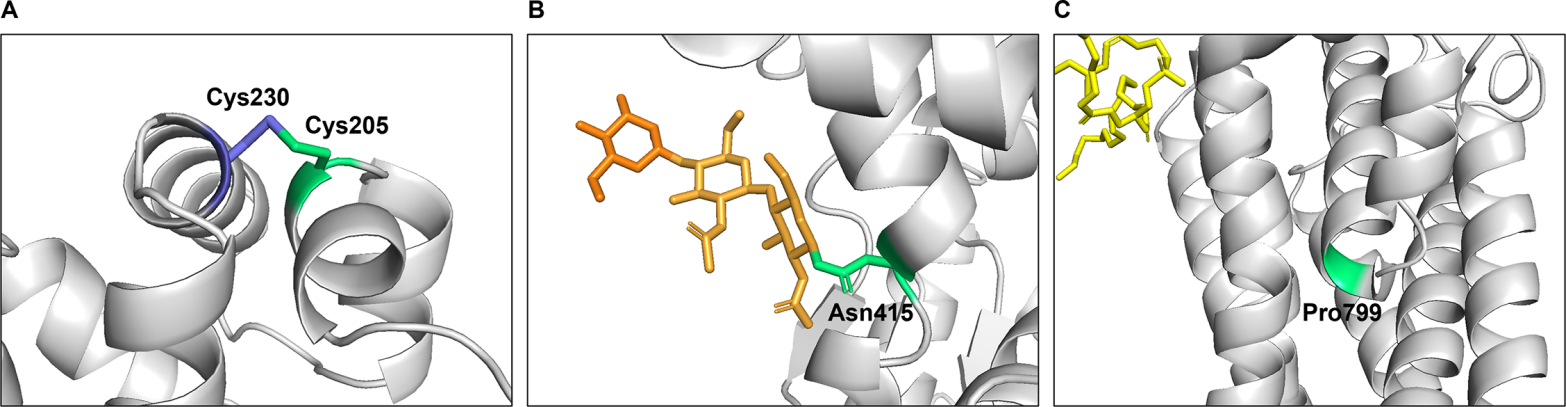
Close-up images of the 3 residues Cys205, Asn415, and Pro799. (**A**) Cys205 (*green*) is located in the ECD1 domain and might form a disulfide bridge with the close cysteine residue at position 230 (*blue*). (**B**) Asn415 (*green*) is located in the ECD1 domain and is post-translationally modified with N-linked glycosylation (*yellow*). (**C**) Pro799 (*green*) is located in the TMD1 domain and causes a kink in the alpha-helix. *N-*Ret-PE (*yellow*) is bound to ABCA4 in the substrate binding pocket.

Two of the variants, Cys205Phe and Asn415Thr, showed severe effects on ABCA4’s function, in which both the basal- and substrate-stimulated activity were reduced. Cys205Phe also showed low solubilization and protein expression level, as well as a tendency to be retained in the ER. In contrast, Asn415Thr showed only a modest decrease in expression and did localize to vesicles. Regarding their F-indexes, both Cys205Phe and Asn415Thr have F-indexes of approximately 0, although the phenotypes of the 2 patients carrying the variants were significantly different. The patient carrying Cys205Phe (P4) had a later age at presentation (36 years) and was diagnosed with ABCA4-RD in stage I, having a non-progressing bull's eye maculopathy without flecks (see Fig. 5A). This mild phenotype is likely attributed to P4’s second variant Gly1961Glu, and correlates well with previous reports on the phenotypes associated with Gly1961Glu.^5^^,^^22^ The variant is a frequent pathogenic variant known to cause a mild outcome irrespective of the severity of the variant in *trans*. Consequently, Gly1961Glu has been described as exhibiting “clinical dominance” and the combined effect of the variant in *trans* does not seem to vary a lot,^6^ a finding also confirmed in our Norwegian patient cohort.^11^ In contrast, the patient carrying Asn415Thr (P5) had an early age at presentation (6 years) and a very severe phenotype with advanced retinal degeneration and is today functionally blind (see Fig. 5B). In addition to Asn415Thr, the patient also carries the pathogenic intronic variant c.5461-10T>C in *trans*. Our group and others have previously demonstrated that c.5461-10T>C leads to splicing defects with exons 39–40 skipping, causing a frameshift and a reduced level of ABCA4 protein.^23^ The combined effect of Asn415Thr and c.5461-10T>C, is most likely the cause of the severe phenotype in this patient. As shown in Figure 1, the 2 severe variants, Cys205Phe and Asn415Thr, are both located in the ECD1. Cysteine residues in the ectodomain of ABCA4 have previously been shown to form disulfide bridges to stabilize the protein folding exposed to the disc lumen's oxidizing environment.^24^^,^^25^ There are in total 14 cysteines in the ectodomains of ABCA4 and 5 disulfide bridges that have so far been resolved, except those predicted between Cys1444 and Cys1455, and Cys230 and Cys205. However, the disulfide-reducing agent DTT used in the ABCA4 cryo-EM structure preparations can disrupt disulfide bonds.^25^ We have shown Cys205Phe to be functionally severe, and pathogenic variants have been reported for Cys230, we believe it is likely that amino acid changes at these two close cysteine positions can disrupt a potential disulfide bond between them (Fig. 7A).^26^^,^^27^ This is probably also the case for other reported disease-causing mutations at cysteine residues (Cys54Tyr, Cys75Gly, Cys519Arg, Cys641Ser, Cys1455Arg, and Cys1490Tyr), supporting the importance of cysteines to stabilize the ECDs. The other functionally severe variant Asn415Thr, is located at 1 of 8 asparagine residues that are post-translationally modified with N-linked glycosylation (Fig. 7B). These modifications have key roles in the folding and stability of ABCA4.^24^^,^^25^ The replacement of asparagine with threonine can therefore disrupt the glycosylation at this residue and cause instability and folding defects affecting both protein expression and function. Based on the functional analyses and the other ACMG-AMP criteria, we could reclassify c.614G>T p.(Cys205Phe) and c.1244A>C p.(Asn415Thr) as pathogenic and likely pathogenic, respectively (see Table 4).

Three variants, Val643Gly, Pro799Leu, and Val1433Ile, had decreased substrate-stimulated activity, although they all showed basal activities comparable to WT. Both Val643Gly and Val1433Ile were expressed at similar level to the WT protein and localized to vesicles, in contrast to Pro799Leu, which was the lowest expressing variant and clearly showed intracellular retention in the ER. Our findings are, therefore, consistent with previous studies showing that low-expressing variants tend to be retained in the ER, whereas normal-expressing variants localize to vesicles.^2^^,^^15^^,^^16^ The low expression level of Pro799Leu may indicate that it is misfolded and aggregates. Based on the F-indexes, all three variants were categorized as functionally moderate variants. Because Pro799Leu had an F-index of 0.17, close to functionally severe, it was expected to cause an earlier age at presentation than Val643Gly (depending on the variant in *trans*). Indeed, the patient carrying Pro799Leu (P7) (see Fig. 6B) had symptoms from the age of 46 years compared to 60 years in the patient carrying Val643Gly (P6) (see Fig. 6A). As shown in Figure 6B, P7 had pathological changes of the retina at the time of diagnosis, however, due to foveal sparing and the slow progressive nature of the disease, the patient was not aware of the loss of visual function before developing diplopia. The earlier age at presentation of P7, compared to P6, is likely also attributed to the second variant c.4734del p.(Leu1580*), a pathogenic nonsense variant. Nonsense, frameshift and splice site variants usually lead to complete loss of function and are often associated with severe degeneration of the retina.^6^ The second variant of P6 is c.6449G>A p.(Cys2150Tyr), which has also previously been classified as pathogenic.

In the case of P10 harboring the Val1433Ile variant, the classification of this patient’s condition as ABCA4-RD was initially uncertain. The clinical description, OCT and UWF-FAF images of P10 (Supplementary Fig. S1B) deviate from the hallmarks of ABCA4-RD, especially the absence of peripapillary sparing.^28^ Instead, the phenotype has some similarities with autosomal dominant Sorsby fundus dystrophy, but the patient has no family history of IRD and the NGS-gene panel containing 334 genes involved in IRD (including *TIMP3*) revealed no disease-causing variant in the *TIMP3* gene. The patient's additional progressive neurological hearing loss raises the suspicion of an underlying ciliopathy, although it is unknown if that is related to the IRD. In addition, we could not detect any other pathogenic *ABCA4* variants in P10, even after analyzing WGS data covering the *ABCA4* locus, except from a rare deep intronic VUS listed in Tables 2 and 4. Therefore, this patient’s staging and disease duration information were not included in Tables 1 and 2.

As shown in Figures 1 and 7C, the residue Pro799 is located in TMD1 where it leads to a kink in the alpha-helix, causing the helix to bend upward in parallel with the other helices in the TMD. The replacement of proline to leucine will most likely disrupt this bending causing an instability of the alpha-helix, which may lead to protein misfolding. This can cause ABCA4 to be more prone to degradation during biosynthetic processing in the ER and can explain the low expression level of Pro799Leu compared to WT. A potential structure instability can also explain the reduced substrate-stimulated ATPase activity of Pro799Leu. We could not predict any structural implications of Val643Gly and Val1433Ile based on the ABCA4 structure model. Although our functional data revealed that these three variants are functionally moderate, we could only reclassify c.2396C>T p.(Pro799Leu) as likely pathogenic, in contrast to c.1928T>G p.(Val643Gly) and c.4297G>A p.(Val1433Ile), which remain VUS. This is due to the high allele frequency of Val643Gly and Val1433Ile in GnomAD. However, both of them have previously been reported as hypomorphic, and therefore we cannot rule out that they might play a role if found in *trans* with a severe loss of function ABCA4 variant.^1^ The ACMG-AMP guidelines that were used for the classification of the variants in the current study are not suitable for hypomorphic variants.^10^

The remaining 5 variants, Tyr106Phe, Gly172Ser, Pro940Arg, Lys1164Arg, and Val2050Leu, did not seem to have any effect on the ABCA4 protein function as their expression levels and ATPase activities were close to normal, confirmed by F-indexes above 0.90. Neither did they affect the intracellular localization of ABCA4 as they all exhibited vesicular distribution. The Tyr106Phe variant was detected in the 2 monozygotic twins, P1 and P2. They also carried 2 other *ABCA4* variants that probably are responsible for their phenotype; c.868C>T p.(Arg290Trp) and c.2875A>G p.(Thr959Ala), now classified as pathogenic and likely pathogenic, respectively. The patient carrying Pro940Arg (P8) was also found to carry 2 other *ABCA4* variants; c.3364G>A p.(Glu1122Lys) classified as pathogenic and c.161-23T>G, classified as likely pathogenic, thus likely accountable for the ABCA4-RD phenotype. The Lys1164Arg variant was detected in P9, who also carries 2 rare deep intronic variants; c.769-784C>T p.(Leu257=, Leu257Aspfs*3) and c.4540-2077C>T. Both intronic variants have previously been reported in patients with suspected ABCA4-RD.^29^^–^^31^ The c.769-784C>T variant has been shown to have a partially deleterious effect on RNA splicing, indicating that some WT protein is still produced.^29^^,^^32^ However, both of these intronic variants are classified as VUS leaving P9 genetically unsolved. The patient carrying Gly172Ser and Val2050Leu (P3) did not carry any other rare *ABCA4* variant. However, further screening of this patient by NGS-retina panel sequencing revealed a homozygous pathogenic *EYS* variant (NM_001142800.2) c.8648_8655del p.(Thr2883Lysfs*4) associated with retinitis pigmentosa 25 (OMIM #602772). UWF-FAF images of P3 (see Supplementary Fig. S1A) demonstrated an annular hyperfluorescent ring and a central hyperfluorescent ring around the fovea. This UWF-FAF pattern, together with loss of color vision, is not typical for ABCA4-RDs. The staging, disease duration and BCVA of this patient are therefore not included in Tables 1 and 2. Based on the functional data of these variants, c.317A>T p.(Tyr106Phe), c.2819C>G p.(Pro940Arg), c.3491A>G p.(Lys1164Arg), and c.6148G>C p.(Val2050Leu) could be reclassified as likely benign. Hence, our finding proves the value of functional characterization for excluding *ABCA4* variants that were originally thought to be associated with the patient's disease. A limitation of our study is that we investigated all the *ABCA4* variants separately and not in combination with the variants found in *cis* in the patients. Our functional analyses may, therefore, not account for any potential *cis*-modifying effects of the investigated variants. We also acknowledge the importance of validating these findings in more relevant retinal cell lines in future studies to strengthen the applicability and relevance of our findings within the retinal context.

We conclude that functional analyses of *ABCA4* missense VUS combined with detailed clinical characterization are highly valuable for variant interpretation and classification of genotypes, leading to a precise diagnosis in patients with suspected ABCA4-RDs.

## Supplementary Material

Supplement 1
