# Supplementary material for: Functional Characterization of ABCA4 Missense Variants Aids Variant Interpretation and Phenotype Prediction in Patients With ABCA4-Retinal Dystrophies
Source: Invest Ophthalmol Vis Sci. 2024 Aug 1;65(10):2. doi: 10.1167/iovs.65.10.2 (PMC11305421; doi:10.1167/iovs.65.10.2)
Supplement: Supplement 1 [file iovs-65-10-2_s001.pdf]

Supplement.

A Patient P3

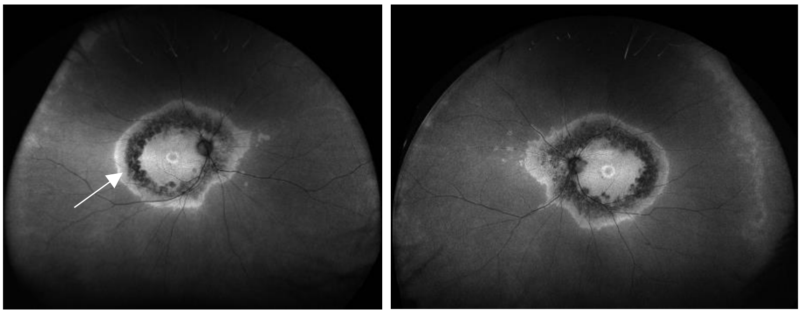

B Patient P10

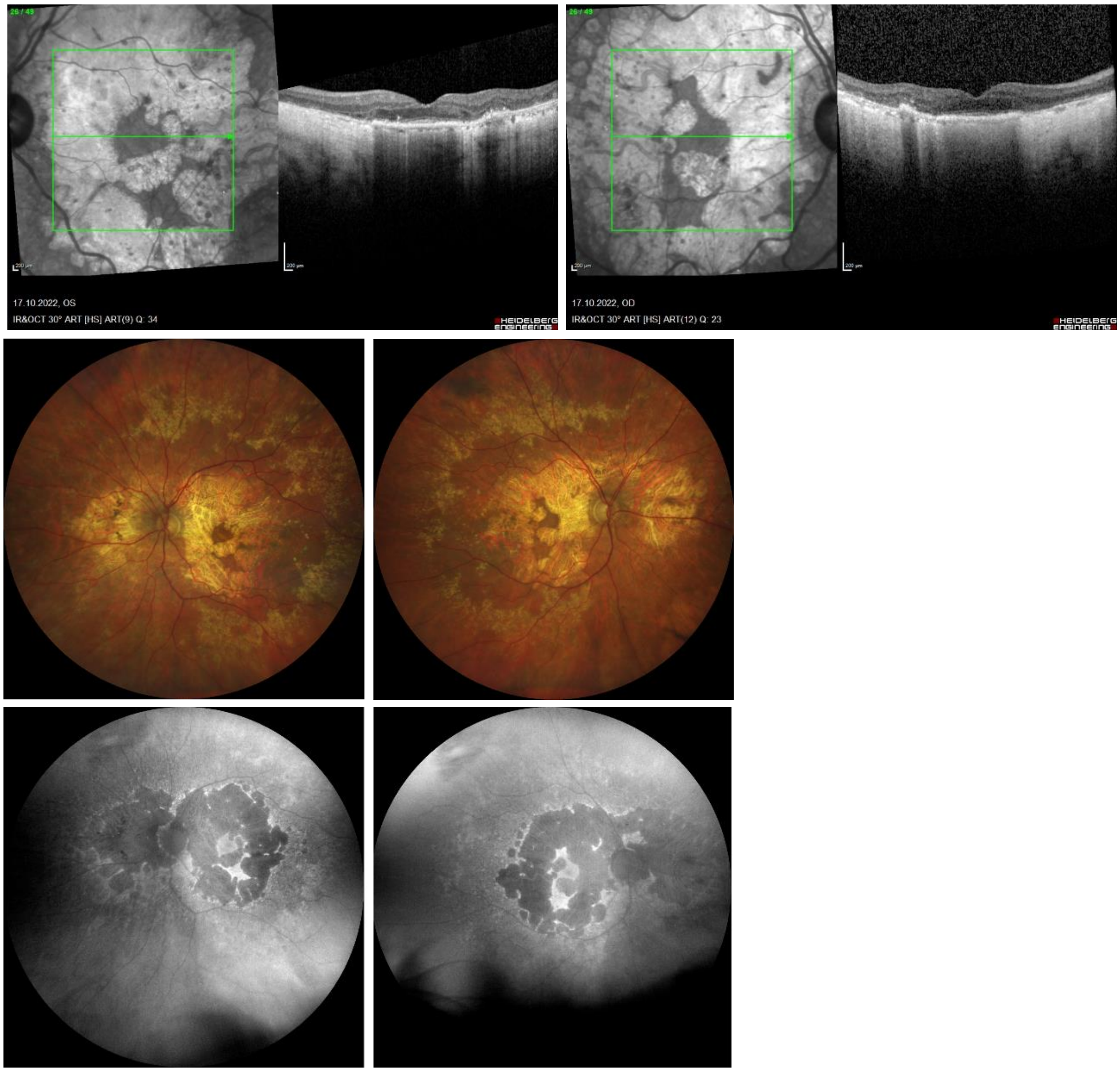

Figure S1. OCT and UWF-FAF images of P3 and P10 with clinical phenotypes deviating from ABCA4-IRD. (A) P3: EYS-mediated cone dystrophy. The hyperfluorescent ring in the midperipheral (marked with a white arrow) area is typical for EYS-mediated disease and not ABCA4-RDs. (B) P10: Patchy and partly confluent atrophy of the posterior pole with central macular sparing and yellow-white midperipheral deposits.
